# Higher than expected CO_2_ fertilization inferred from leaf to global observations

**DOI:** 10.1111/gcb.14950

**Published:** 2020-02-04

**Authors:** Vanessa Haverd, Benjamin Smith, Josep G. Canadell, Matthias Cuntz, Sara Mikaloff‐Fletcher, Graham Farquhar, William Woodgate, Peter R. Briggs, Cathy M. Trudinger

**Affiliations:** ^1^ CSIRO Oceans and Atmosphere Canberra ACT Australia; ^2^ Department of Physical Geography and Ecosystem Science Lund University Lund Sweden; ^3^ Hawkesbury Institute for the Environment Western Sydney University Penrith NSW Australia; ^4^ AgroParisTech Université de Lorraine INRA UMR Silva Nancy France; ^5^ National Institute of Water and Atmospheric Research Wellington New Zealand; ^6^ Research School of Biology The Australian National University Canberra ACT Australia; ^7^ CSIRO Land & Water Canberra ACT Australia; ^8^ CSIRO Oceans and Atmosphere Melbourne Vic. Australia

**Keywords:** amplitude of seasonal cycle, carbonyl sulfide, CO_2_ fertilization, coordination of photosynthesis, gross primary production, land carbon sink

## Abstract

Several lines of evidence point to an increase in the activity of the terrestrial biosphere over recent decades, impacting the global net land carbon sink (NLS) and its control on the growth of atmospheric carbon dioxide (*c_a_*). Global terrestrial gross primary production (GPP)—the rate of carbon fixation by photosynthesis—is estimated to have risen by (31 ± 5)% since 1900, but the relative contributions of different putative drivers to this increase are not well known. Here we identify the rising atmospheric CO_2_ concentration as the dominant driver. We reconcile leaf‐level and global atmospheric constraints on trends in modeled biospheric activity to reveal a global CO_2_ fertilization effect on photosynthesis of 30% since 1900, or 47% for a doubling of *c_a_* above the pre‐industrial level. Our historic value is nearly twice as high as current estimates (17 ± 4)% that do not use the full range of available constraints. Consequently, under a future low‐emission scenario, we project a land carbon sink (174 PgC, 2006–2099) that is 57 PgC larger than if a lower CO_2_ fertilization effect comparable with current estimates is assumed. These findings suggest a larger beneficial role of the land carbon sink in modulating future excess anthropogenic CO_2_ consistent with the target of the Paris Agreement to stay below 2°C warming, and underscore the importance of preserving terrestrial carbon sinks.

## Graphical abstract

CO_2_ concentrations in the global atmosphere will continue to rise for decades ahead. Plants grow faster under elevated CO_2_, but the current generation of carbon cycle models, including those embedded in Earth system models used for climate assessment, diverge in their representation and future projections of this response. By invoking long‐standing theory on how plants use a limited supply of nitrogen in order to optimize photosynthesis and growth, we reconcile leaf‐level and recent global atmospheric constraints on CO_2_ fertilization.

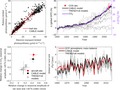

## 1 INTRODUCTION

Four independent lines of evidence indicate intensifying terrestrial biosphere activity (Campbell et al., 2017; Forzieri, Alkama, Miralles, & Cescatti, 2017; Graven et al., 2013; Le Quéré et al., 2018), namely the increasing positive trends in (a) the global net land carbon sink (NLS; Jinfeng et al., 2017; Le Quéré et al., 2018; Li et al., 2018); (b) the amplitude of the seasonal cycle (ASC) of atmospheric CO_2_ (*c_a_*) in the Northern Hemisphere (Forkel et al., 2016; Graven et al., 2013; Thomas et al., 2016); (c) satellite‐observed leaf area (Forzieri et al., 2017; Zhu et al., 2016); and (d) global gross primary production (GPP), as inferred from long‐term atmospheric carbonyl sulfide concentration (COS) records (Campbell et al., 2017). Attribution and future projection of these trends relies on terrestrial biosphere models (TBMs) that encapsulate mechanistic understanding of the terrestrial carbon cycle and are used to inform the global carbon budget (Le Quéré et al., 2018; Sitch et al., 2015).

Our focus in this work is on the historical increase in global GPP. The estimate presented by Campbell et al. (2017) [(31 ± 5)% increase (2σ, 1900–2010)] is based on the long‐term atmospheric carbonyl sulfide (COS) records, derived from ice‐core, firn and ambient air samples. Campbell et al. (2017) interpreted these records using a model that simulates the changes in atmospheric COS concentration according to the changes in its sources and sinks—including a large sink that is related to GPP—and suggested that their results provide a global‐scale benchmark for historical carbon cycle simulations. The accuracy of COS‐based GPP estimates depends in part on accounting for different environmental responses of COS and CO_2_ uptake (Whelan et al., 2018). Despite recent progress in accounting for these different responses (Kooijmans et al., 2019), the estimate by Campbell et al. (2017) remains the only COS‐based estimate of the historical change in global GPP and has yet to be utilized as a benchmark for global TBMs. Previous estimates of the global GPP increase over this timescale are restricted to process model simulations that suggest a much lower increase of 16 ± 4% (Keenan et al., 2016). While there is consensus that the simulated increase is largely driven by CO_2_ fertilization (Baldocchi, Ryu, & Keenan, 2016; Keenan et al., 2016; Schimel, Stephens, & Fisher, 2015), the general underprediction of the global GPP increase means that a large fraction (>one‐third) of the 31% ± 5% growth is unaccounted for by current TBMs, impacting their future projections of the dynamics of the NLS. Furthermore, it remains to be explored whether the large COS‐based increase in global GPP is compatible with the other lines of evidence for increased terrestrial biospheric activity, particularly trends in the NLS and ASC.

The NLS may be estimated as the sum of atmosphere and ocean sinks minus the emissions from cement productions and combustion of fossil fuels. The NLS is positive (2.3 ± 0.4 PgC/year; 2007–2016) and has been increasing at a rate of 0.061 ± 0.02 (1 *SE*) PgC/year^2^ (1980–2016; Le Quéré et al., 2018), implying that, globally, GPP is in excess of ecosystem carbon loss by fire and respiration, and that GPP is increasing faster than ecosystem carbon loss. As such, the magnitude and trend in the NLS provide indirect constraints on the magnitude of global GPP increase. Interannual variations in the NLS—driven largely by anomalies in plant productivity (Ahlström et al., 2015)—also provide a constraint on the sensitivity of simulated productivity to temperature and precipitation anomalies (Piao et al., 2013).

The ASC in the Northern Hemisphere (averaged over latitudes >45°N, 500 mb), as calculated from aircraft data in two separate 3‐ to 4‐year periods (IGY data; 1958–1961; Keeling, Harris, & Wilkins, 1968) and (HIPPO and NOAA data; 2009–2011; Wofsy, 2011), has been reported to show a large increase of (57 ± 7)% (Graven et al., 2013). Atmospheric transport simulations reveal that these ASC observations are sensitive to net uptake on land north of 40°N, with negligible contributions from lower latitudes or from the ocean (Graven et al., 2013). The increase in ASC in studies examining the multi‐model TBM simulations combined with atmospheric transport modeling show that the simulated increase in seasonal amplitude of *c_a_* is generally underpredicted both at high altitude (Graven et al., 2013) and at remote surface stations (Thomas et al., 2016). It has been speculated that this is because TBMs underpredict CO_2_ and/or warming effects on GPP (Forkel et al., 2016; Thomas et al., 2016), but changes in the seasonal cycle of ecosystem respiration (e.g., Liptak, Keppel‐Aleks, & Lindsay, 2017), including winter respiration (Parazoo et al., 2016), must also be implicated.

**Figure 1:**
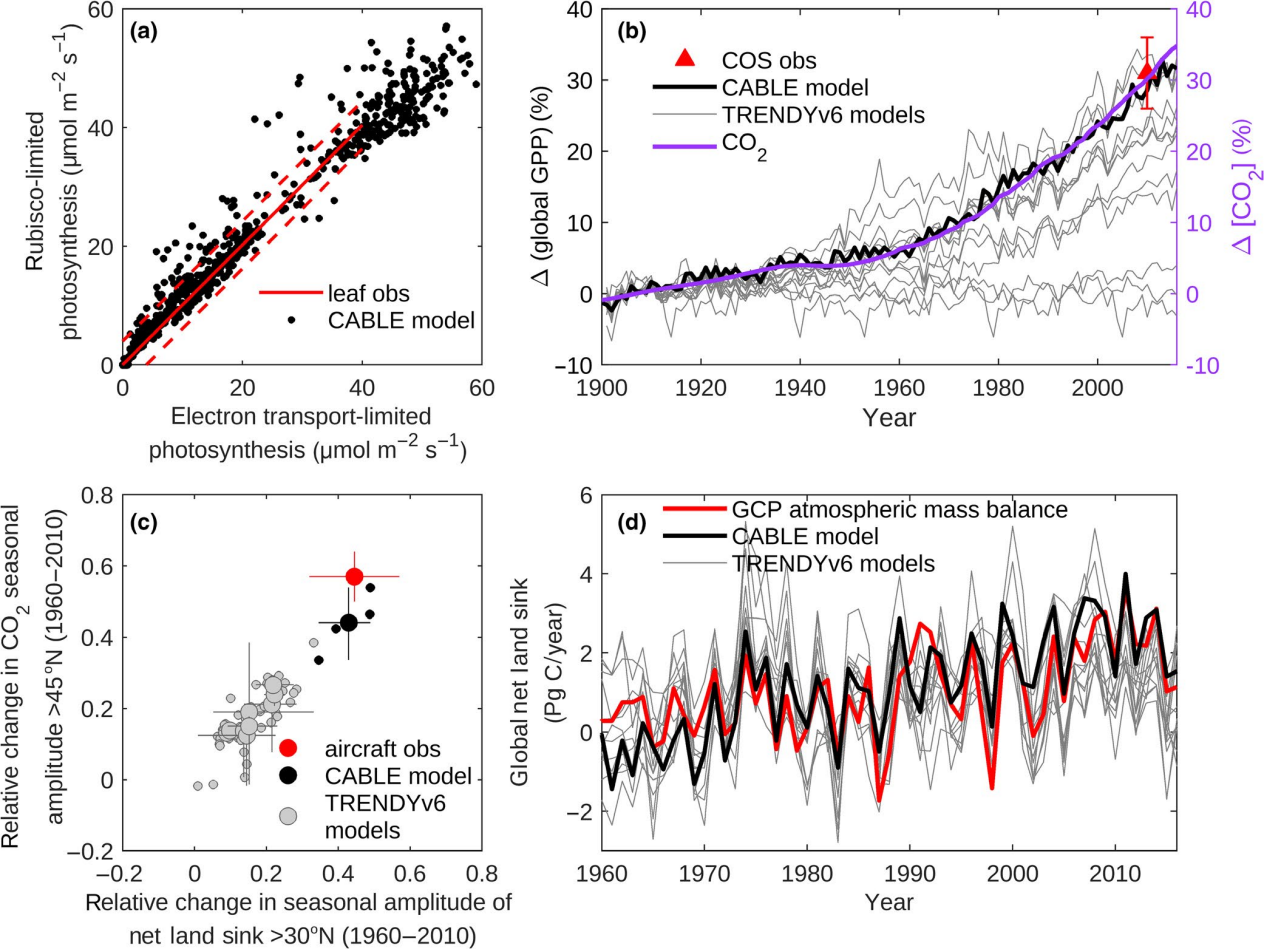
Constraints on trends in global‐scale terrestrial biospheric activity. (a) Coordination of photosynthesis. Black: CABLE predictions of *A_c_* versus *A_j_* contributions to C_3_ photosynthesis (1980–2015) at 1,000 randomly distributed grid cells across the global ice‐free land surface. Red lines reproduced from Maire et al. (2012): *A_c_* versus *A_j_* under average plant growth conditions predicted from observed leaf‐level traits (solid); predictive error (dashed). (b) Increase in global gross primary production relative to 1900, simulated by CABLE (black) and TRENDYv6 (Le Quéré et al., 2018; Sitch et al., 2015 grey), and inferred from observations of [COS] (4) (red); Increase in *c_a_* (purple; Dlugokencky & Tans, 2017). (c) Increase in amplitude of the seasonal cycle (ASC) of *c_a_* (1958–1961 to 2009–2011, >45°N, 500 mb) versus ASC of net land carbon sink (NLS) north of 30°N: aircraft observations (Keeling et al., 1968; Wofsy, 2011) and corresponding TM3 (Heimann & Körner, 2005) atmospheric inversion reproduced from Graven et al. (2013; red); CABLE‐TM3 (black); TRENDYv6‐TM3 (grey); average for each model (large circles); individual ensemble members (small circles); ensemble range (bars; Section 2). (d) Time‐series of annual global NLS: CABLE (black); TRENDYv6 (grey); Global Carbon Project (GCP) mass balance estimate (note 0.4 PgC/year uncertainty), as the sum of atmosphere and ocean sinks, minus fossil fuel emissions (red). Linear trends (1980–2015): 0.061 ± 0.02 (1 *SE*) PgC/year^2^ (GCP) and 0.067 ± 0.01 PgC/year^2^ (CABLE). See also Figure S1 for metrics related to the NLS

The magnitude of the CO_2_ fertilization effect on photosynthesis depends on biochemical demand for CO_2_ and supply of CO_2_ from the atmosphere to the chloroplast via the stomata. Global TBMs typically simulate biochemical demand by C_3_ plants (accounting for ~77% of photosynthesis globally; Still, Berry, Collatz, & Defries, 2003) using the photosynthesis model of Farquhar and von Caemmerer (1981), which equates gross assimilation (*A*) with the lesser of Rubisco and electron transport‐limited assimilation rates (*A_c_* and *A_j_,* respectively). The sensitivities of *A_c_* and *A_j_* to *c_a_* are well known (Lloyd & Farquhar, 1996; Long, 1991) and quite different, meaning that the relative contributions of *A_c_* (high sensitivity) and *A_j_* (low sensitivity)‐limited rates to *A_n_* are an important determinant of the leaf‐level CO_2_ fertilization effect. At the leaf level, the dimensionless elasticity of gross carbon assimilation by photosynthesis (*A*) to *c_a_* $\left(\beta =\frac{\partial A}{\partial c_{a}}\frac{c_{a}}{A}\right)$ characterizes the instantaneous sensitivity of *A* to *c_a_*. Leaf gas‐exchange observations (Farquhar & von Caemmerer, 1981; Maire et al., 2012) support the coordination theory (Chen, Reynolds, Harley, & Tenhunen, 1993; Farquhar & von Caemmerer, 1981; Wang et al., 2017) that plants optimize the productivity in their environment through relative nitrogen investment in electron transport and Rubisco‐limited steps in the photosynthesis chain, such that they are co‐limiting. Such co‐limitation occurs for average growing conditions spanning wide‐ranging *c_a_*, vapor pressure deficit, light, and temperature (Maire et al., 2012), with the result that the long‐term contributions of *A_c_* and *A_j_* to *A* are approximately equal. The results of Maire et al. (2012; reproduced in Figure 1a) represent the predictions of *A_c_* and *A_j_* for mean growth conditions of the previous month, based on the experimental values of photosynthetic leaf traits. The response to increasing *c_a_* appears to be most marked, with a reduction in Rubisco content of leaves, in plants growing in nutrient‐limited environments (Wong, 1979). This coordination of photosynthesis, and its associated constraint on *β*, is not generally represented in TBMs, although approaches are emerging that enable such representation (Ali et al., 2016; Haverd et al., 2018; Wang et al., 2017). The usefulness of this constraint on CO_2_ fertilization as a driver of global GPP variation has so far been largely neglected.

Here we reconcile the leaf‐level constraint on the CO_2_ fertilization effect imposed by coordination of photosynthesis with the constraints implied by trends in biospheric activity reflected in globally observed increases in ASC, NLS, and GPP inferred from atmospheric COS. This is achieved using mechanistic principles encapsulated by a TBM framework (Pugh et al., 2016) that links biophysics, ecophysiology, biogeochemistry (including nitrogen cycle), and vegetation structural dynamics via dependencies on climate, *c_a_*, disturbance and land‐use drivers, and evolving system state. We quantify the contribution of the biospheric CO_2_ response to the trend in global GPP and explore the potential implications for the future evolution of the NLS.

## 2 MATERIALS AND METHODS

### 2.1 Community Atmosphere‐Biosphere Land Exchange

The Community Atmosphere‐Biosphere Land Exchange model (CABLE) is a biogeochemical land surface model (including N‐cycle) that represents vegetation structural dynamics. CABLE consists of a biophysics core (Haverd, Cuntz, Nieradzik, & Harman, 2016; Kowalczyk et al., 2013; Wang et al., 2011), the CASA‐CNP biogeochemistry module (Wang, Law, & Pak, 2010), the POP module for woody demography and disturbance‐mediated landscape heterogeneity (Haverd, Smith, et al., 2013), and a module for land use and land management (POPLUC; Haverd et al., 2018). The land use and land cover change module is driven by gross land use transitions and wood harvest area. CABLE is one of an ensemble of ecosystem and land surface models contributing to the Global Carbon Project's annual update of the global carbon budget (Le Quéré et al., 2018) via the TRENDY ensemble.

This article uses a version of CABLE that was recently extended to account for (a) tree demography and disturbance‐mediated landscape heterogeneity; (b) land use and land cover change; and (c) coordinated leaf nitrogen investment in electron transport and Rubisco‐limited steps in the photosynthesis chain, such that they adjust seasonally and across biomes to be co‐limiting (Haverd et al., 2018). This version also includes a new diagnostic elasticity $\beta =\left(\partial A/\partial c_{s}\right)\left(c_{s}/A\right)$ that allows the GPP increase attributable to the leaf‐level component of the CO_2_ fertilization effect to be isolated as $\left(\partial A/\partial c_{s}\right)\Delta c_{s}$, where *c_s_* is the CO_2_ concentration at the leaf surface.

### 2.2 CABLE: Coordination of C_3_ photosynthesis

CABLE represents the coordination of C_3_ photosynthesis, adjusting electron transport and Rubisco‐limited rates (Farquhar, Caemmerer, & Berry, 1980) to be co‐limiting (Haverd et al., 2018). This is achieved by dynamically optimizing the ratio *b_JV_* of the maximum rate of electron transport (*J*_max_) to the Rubisco‐catalyzed maximum rate of carboxylation (*V*_cmax_) at standard temperature. The optimization aims to maximize the rate of photosynthesis per unit leaf nitrogen. The approach to optimization of *b_JV_* is based on the following three assumptions:

1. Leaf nitrogen resources may be dynamically redistributed at a 5‐day timescale at no cost, that is *b_JV_* is optimized, such that net photosynthesis (given total available leaf nitrogen) accumulated over the last 5 days (approximately the timescale for turnover of photosynthetic nitrogen; Thornley, 2004) would have been maximized.
2. Leaf nitrogen resources available for partitioning between Rubisco and electron transport capacity are proportional to effective nitrogen content (*N*_eff_), defined as the sum of prior estimates of *V*_cmax,0_ and *J*_cmax,0_, weighted by relative cost factor *c*_cost,_ *_JV_*. (There are no explicit nitrogen pools corresponding to Rubisco and electron transport capacities because there are no explicit nitrogen pools in the photosynthesis model of Farquhar et al. 1980.)
3. The prior values of *V*_cmax,0_ (related to time‐varying prognostic leaf nitrogen content) and *b_JV_* are prescribed according to the synthesis of globally distributed leaf gas‐exchange measurements by Walker et al. (2014).
  The method for implementing these assumptions in CABLE is:

1. Maintain a 5‐day history of sub‐diurnal leaf‐level meteorology (absorbed PAR; leaf‐air vapor pressure difference; leaf temperature, CO_2_ concentration at the leaf surface) for sunlit and shaded leaves, such that total photosynthesis over the last 5 days can be reconstructed.
2. Construct a function that calculates leaf nitrogen cost per unit net photosynthesis. Inputs to this function are as follows: (a) current estimate of *b_JV_*, (b) effective leaf nitrogen content (related to prior value of *V*_cmax,0_), (c) 5‐day history of sub‐diurnal leaf‐level meteorology.
3. At the end of each day, solve for *b_JV_* that maximizes the total photosynthesis over the last 5 days per unit effective leaf nitrogen, and update *V*_c,max,0_ and *J*_max,0_ values.
  The emerging contributions of electron transport and Rubisco‐limited rates contribute approximately equally to total net photosynthesis.

### 2.3 Ensemble of current TBMs

We use simulations from the TRENDYv6 ensemble of TBMs to represent current TBMs: these models (Table S1) encapsulate mechanistic understanding of the terrestrial carbon cycle and are used to inform the global carbon budget. Simulations generated using common protocols and forcings are made available at monthly time resolution and variable spatial resolution for the time period 1860–2016. We used estimates of the simulated annual global NLS (Figure 1d) directly from the Global Carbon Budget 2017 (42). Gridded TRENDY outputs (GPP and NLS) were downloaded from http://sftp://trendy-v6:gcp-2017@emps-cpdn.ex.ac.uk/output/ (accessed September 21, 2017).

### 2.4 CABLE: Historic simulations

Global simulations were performed at 0.5 × 0.5° spatial resolution, with time steps of 3 hr (biophysics); 1 day (biogeochemistry); and 1 year (woody demography, disturbance, land use change). The nitrogen cycle was enabled, but not the phosphorus cycle. Recently developed parameterizations for drought response of stomatal conductance and effects of leaf litter on soil evaporation were enabled (Haverd et al., 2016). The relationship between *V*_c,max,0_ and leaf nutrient status was prescribed using the meta‐analysis of globally distributed leaf gas‐exchange data by Walker et al. (2014). Other photosynthetic parameters were prescribed to be consistent with this analysis, in which any effect of mesophyll conductance (*g*_m_) is implicit in the inferred, effective value of *V*_cmax,0_. This effective value will be lower than the true Rubisco capacity owing to the resistance to gas transport between the stomatal chamber and the chloroplast stroma where carboxylation takes place. Following the recent recommendation of Bahar, Hayes, Scafaro, Atkin, and Evans (2018): “while incorporating *g*_m_ into models to estimate GPP is theoretically appealing, in practice it is not yet possible as it requires knowledge of Rubisco kinetic parameters for a range of evergreen tree species, an estimate of *g*_m_ and its temperature response for representative species,” we chose not to account for *g*_m_ explicitly.

Simulations were driven by (a) daily CRU‐NCEP V7 (1901–2016; Viovy, 2009), down‐scaled to 3‐hourly resolution using a weather generator (Haverd, Raupach, et al., 2013); (b) CO_2_ (1‐year) resolution (Dlugokencky & Tans, 2017); (c) gridded nitrogen deposition (10‐year resolution; Lamarque et al., 2011); and (d) gridded gross land use transitions and harvest (1500–2015) and initial land use states (1,500) from the LUH2 harmonized land use data set (Hurtt et al., 2011; Hurtt, Chini, Frolking, & Sahajpal, 2016).

The “Excl. Coord.” configuration of the model that excluded the coordination of C_3_ photosynthesis differed from the full model in three ways: (a) dynamic optimization of the *J*_max,0_ */V*_cmax,0_ ratio was disabled; (b) the *J*_max,0_ */V*_cmax,0_ ratio was set to a low value of 1.3 to ensure that *A_J_* was always less than *A_c_*; (c) *V*_cmax,0_ was scaled up by a factor of 1.25 to ensure that the simulated present day GPP from this configuration and the full model configuration are approximately equal (Figure S3a).

**Figure 2:**
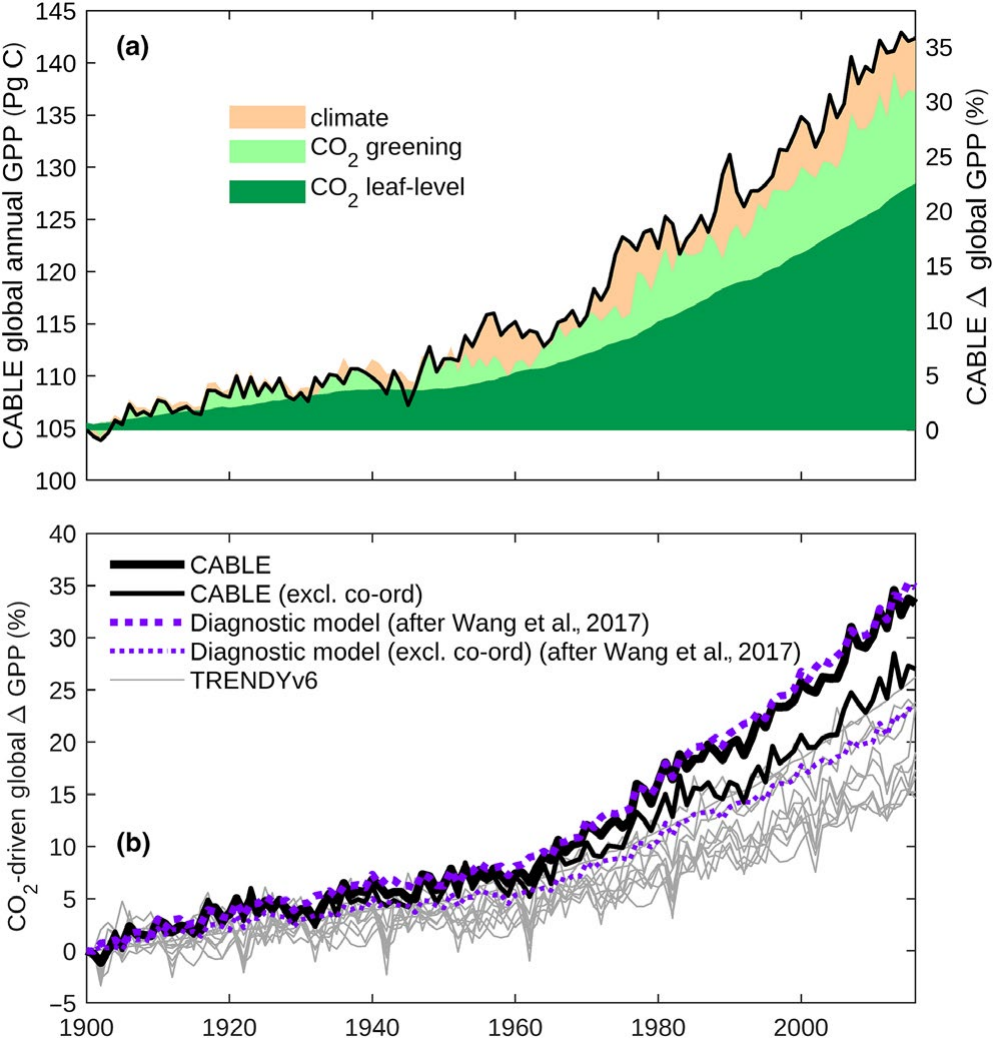
CO_2_ fertilization effect on global gross primary production (GPP). (a) Full CABLE simulation with dynamic land use (solid black lines) attributed to: (i) climate effect and *c_a_* × climate interaction effect (orange); (ii) CO_2_ greening effect (light green); (iii) CO_2_ leaf‐level effect (dark green). (b) CO_2_‐driven increase in global GPP relative to 1900 (fixed 1,860 land use), as simulated by: (i) full CABLE (thick black line); (ii) CABLE without coordination of photosynthesis, that is assimilation is always limited by electron transport (thin black line); (iii) the TRENDYv6 ensemble (grey lines) and (iv) full diagnostic photosynthesis algorithm of Wang et al. (2017), applied using prescribed vegetation cover from CABLE (thick purple dashed line); (v) diagnostic photosynthesis algorithm of Wang et al. (2017) without coordination (i.e., assimilation is always limited by electron transport), applied using prescribed vegetation cover from CABLE (thin purple‐dashed line)

The partitioning of CABLE’s historic global GPP between climate and CO_2_ effects (Figure 2a; Figure S5) was achieved by subtracting the results of a “CO_2_ only experiment” from an “all driver experiment.” In the CO_2_‐only experiment, CO_2_ and land use are varied and recycled meteorology (1901–1930) is prescribed.

In contrast, the CO_2_‐driven increase in GPP is derived using the “S1” scenario from the TRENDY protocol, in which fixed 1,860 land cover is assumed and only CO_2_ is varied. This additional simulation allows for direct comparison of the simulated CO_2_ fertilization effect with the TRENDYv6 ensemble.

We performed further single and double‐factorial experiments to isolate the effects of single drivers and driver combinations: *c_a_* only; temperature only; *c_a_* and temperature together; precipitation; land use change.

### 2.5 CABLE: Future projections

The Representative Concentration Pathways (RCPs) were designed to inform the mitigation and adaptation policies, and describe different climate futures based on a range of emissions scenarios (van Vuuren et al., 2011). The Shared Socioeconomic Pathways (SSPs) consist of trajectories of future human development with different socioeconomic conditions and associated land use projections (O’Neill et al., 2017). The SSPs can be combined with the RCPs to explore a range of futures for climate change and land use change. We used SSP1 combined with RCP2.6 (low conversion of land use and low emissions) to project the NLS to 2006–2099 (Figure 5). This simulation was forced using harmonized historic and future data sets for climate (IPSL‐CM5A‐LR from ISI‐MIP2a; Warszawski et al., 2014; https://esg.pik-potsdam.de/search/isimip2a/, accessed December 19, 2017), land use (Hurtt et al., 2016; Hurtt, Chini, Frolking, & Sahajpal, 2018), CO_2_ (van Vuuren et al., 2011), and nitrogen deposition (Lamarque et al., 2011). We performed two simulations: (a) full simulation with all drivers varying to give the NLS and (b) a simulation of the biophysical sink (i.e., the net carbon uptake driven by changing climate, CO_2_, and nitrogen deposition) for each land cover class, with class contributions weighted by their time‐varying area fractions.

To assess the CO_2_ fertilization effect on GPP for a doubling of *c_a_*, we also performed a projection (2006–2099) using CO_2_ forcing from RCP8.5, combined with fixed IPSL‐CM5A‐LR climate (repeated 1990–2010) and land cover (2015). The simulated trend in GPP over this period corresponds to projected CO_2_ fertilization effects of (47 ± 2)% for a doubling of *c_a_* (300–600 ppm, constant climate and land use) for the globe and (42 ± 2)%, for the Northern Hemisphere extra‐tropics >30°N.

#### 2.5.1 Amplitude of CO_2_ seasonal cycle in the Northern Hemisphere

The increase in amplitude of seasonal cycle in *c_a_* was simulated by using air–land fluxes from the CABLE and TRENDYv6 TBMs as boundary conditions for “fine grid” version of the global atmospheric tracer transport model TM3 (Heimann & Körner, 2005), with an approximate horizontal resolution of 3.8 × 5° and 19 vertical levels. It was driven by winds from NCEP reanalysis (Kalnay et al., 1996). Influences from outside of the terrestrial biosphere on seasonal CO_2_ amplitude in the NH, including fossil fuel emissions and air–ocean fluxes have been shown to be negligible (Graven et al., 2013), and were omitted in this analysis. The simulated monthly time series corresponding to each 3‐ to 4‐year aircraft campaigns was interpolated to the 500 mb isobar and linearly detrended. The seasonal CO_2_ amplitude was quantified by taking the difference of the maximum and minimum monthly detrended concentrations.

The NLS in the Northern Hemisphere is the net sum of two similarly large flux components (GPP and ecosytem carbon loss—largely ecosystem respiration, but also including fire loss—to the atmosphere: $R_{\text{eco}}^{\prime }=\text{GPP}-\text{NLS}$) which both have summer seasonal peaks. Poor predictions of the ASC magnitude may result from small errors in the relative phase and/or the amplitude of either flux, which in turn may influence the simulated ASC trends. To address this source of uncertainty, we made the assumption that the seasonal amplitude in GPP is well‐simulated (as confirmed by comparison with latitudinally averaged upscaled FluxNET data (Jung et al., 2011)), but that the relative phase of $R_{\text{eco}}^{\prime }$ may vary by ±14 days and the amplitude by ±20% compared with the simulated values. Such errors in the phase and amplitude of simulated $R_{\text{eco}}^{\prime }$ may arise from a number of factors such as: (a) approximating soil carbon stocks as pools with discrete turnover times and associated effective soil depths (Koven et al., 2013), (b) neglecting seasonal acclimation effects on autotrophic and heterotrophic respiration. Under this assumption, we constructed 25 ensembles of monthly gridded NLS for CABLE and for each model of the TRENDYv6 ensemble for which monthly output could be obtained. Each ensemble member corresponded to one of five equally spaced phase shifts and amplitude scalings of $R_{\text{eco}}^{\prime }$, while conserving the total annual flux. The ensembles were then used to generate 25 estimates of monthly *c_a_* for Point Barrow *(BRW* 71.3°N, 156.6°W) and Cape Kumukahi (KMK, 19.5°N, 155.6°W), marine boundary layer stations examined by Wenzel, Cox, Eyring, and Friedlingstein (2016). Observed monthly mean in situ atmospheric CO_2_ concentrations at BRW and KMK were obtained from the Scripps Institute of Oceanography records (http://scrippsco2.ucsd.edu/data/atmospheric_co2; Keeling et al., 2001). The ensemble members corresponding to each TBM were selected such that perturbed ASC (2010) at both stations matched the actual ASC to within ±3 ppm. These selected subsets were then used as input to TM3 to simulate the relative change in ASC, with mean and uncertainties for each model expressed as the mean and range of ensemble members (Figure 1c).

## 3 RESULTS

**Figure 3:**
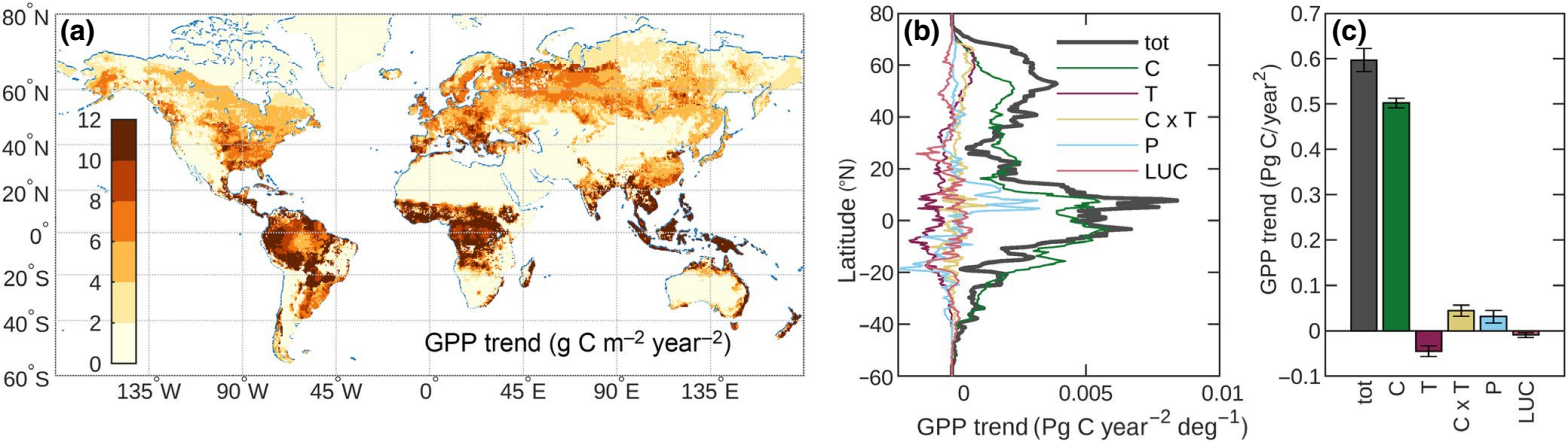
Spatial and driver attribution of gross primary production (GPP) trend (1980–2016). (a) Spatial distribution. (b) Latitudinal distribution of driver components. (c) Driver attribution of global GPP trend. Driver attribution is achieved by factorial experiments in which only one or a pair of drivers is varied while all others are fixed (Section 2). Tot = total effect; C = *c_a_* only; T = temperature; C × T = *c_a_*–temperature interaction; P = precipitation; LUC = land use change. Only major drivers are isolated. Additional drivers (nitrogen deposition; specific humidity; wind speed; incoming short and longwave radiation) and their interactions are not isolated but may contribute to the total effect. Error bars represent standard errors from linear regression fits to each driver component (Section 2)

Using the CABLE TBM, enabled to represent the coordination of photosynthesis (Haverd et al., 2018; Figure 1a), we successfully capture four major independent global‐scale benchmarks of trends in biospheric activity. They are as follows: (a) multi‐decadal increases in global GPP (Figure 1b), (b) amplitude of the seasonal CO_2_ cycle in the Northern Hemisphere (Figure 1c), (c) global NLS (Figure 1d) including its mean, trend, and interannual variations that are considered a proxy for tropical temperature sensitivity (Figure S1), and (d) leaf area index (Figure S2). Using model experiments to attribute the effects of changing climate and *c_a_* (Section 2), we find that the total increase in GPP is predominantly driven by CO_2_ (Figure 2a). The remaining climate‐driven component (14% of the full increase, Figure 2a) is largely attributable to warming and (temperature × *c_a_*) interactions in Northern Hemisphere extratropical ecosystems (Figure 3c; Figure S5).

We estimate a historic global CO_2_ fertilization effect on photosynthesis of 30% (1900–2010; 296–389 ppm c_a_) that is significantly higher than current TBM (TRENDYv6) estimates (17 ± 4%; 1σ), Figure 2b. We project a GPP increase of (47 ± 2)% for a doubling of *c_a_* (300–600 ppm, constant climate and land use, Section 2). Our corresponding extratropical Northern Hemisphere estimate (>30°N) of (42 ± 2)% (1 *SE*) is at the high end of the previously reported estimate (32 ± 9)% (2σ) obtained by constraining an ensemble of climate‐carbon cycle model projections with observed trends in the ASC of *c_a_* (Wenzel et al., 2016).

The simulation of coordination of photosynthesis contributes strongly to the high‐CO_2_ fertilization effect in CABLE (Figure 2b). Both CABLE configurations agree well with present‐day GPP estimates based on upscaled eddy flux data (Jung et al., 2011) in terms of magnitude and latitudinal distribution (Figure S3b), highlighting that the contrasting CO_2_ elasticities (Figure S3a) lead to different magnitudes and latitudinal distributions of the NLS (Figure S3a,c).

We partition the CO_2_ fertilization effect on GPP between leaf‐level and greening effects, using the dimensionless *β* diagnostic, which expresses the instantaneous sensitivity of photosynthesis to CO_2_. This allows the contribution of greening (i.e., increased light harvesting capacity due to the investment of additional plant carbon in a higher leaf area) to be calculated as the difference between overall GPP increase and the leaf‐level contribution to that increase. We find that 70% of the CO_2_‐driven increase in global GPP is attributable to the leaf‐level effect, that is the CO_2_‐induced increase in GPP that occurs at constant leaf area. The remaining 30% is attributable to the CO_2_‐greening effect (Figure 2a), that is the positive feedback resulting from the investment by vegetation of a proportion of increased productivity in enhanced leaf area. The leaf‐level effect from rising *c_a_* includes both the direct biochemical effect (effect of *c_a_* on Rubisco carboxylase as a substrate and inhibition of ribulose bisphosphate oxygenation and hence photorespiration; Long, 1991) and the indirect effect of enhanced water‐use efficiency resulting from the reductions in stomatal conductance that adjusts to maximize carbon gain with respect to water loss (Franks et al., 2013).

**Figure 4:**
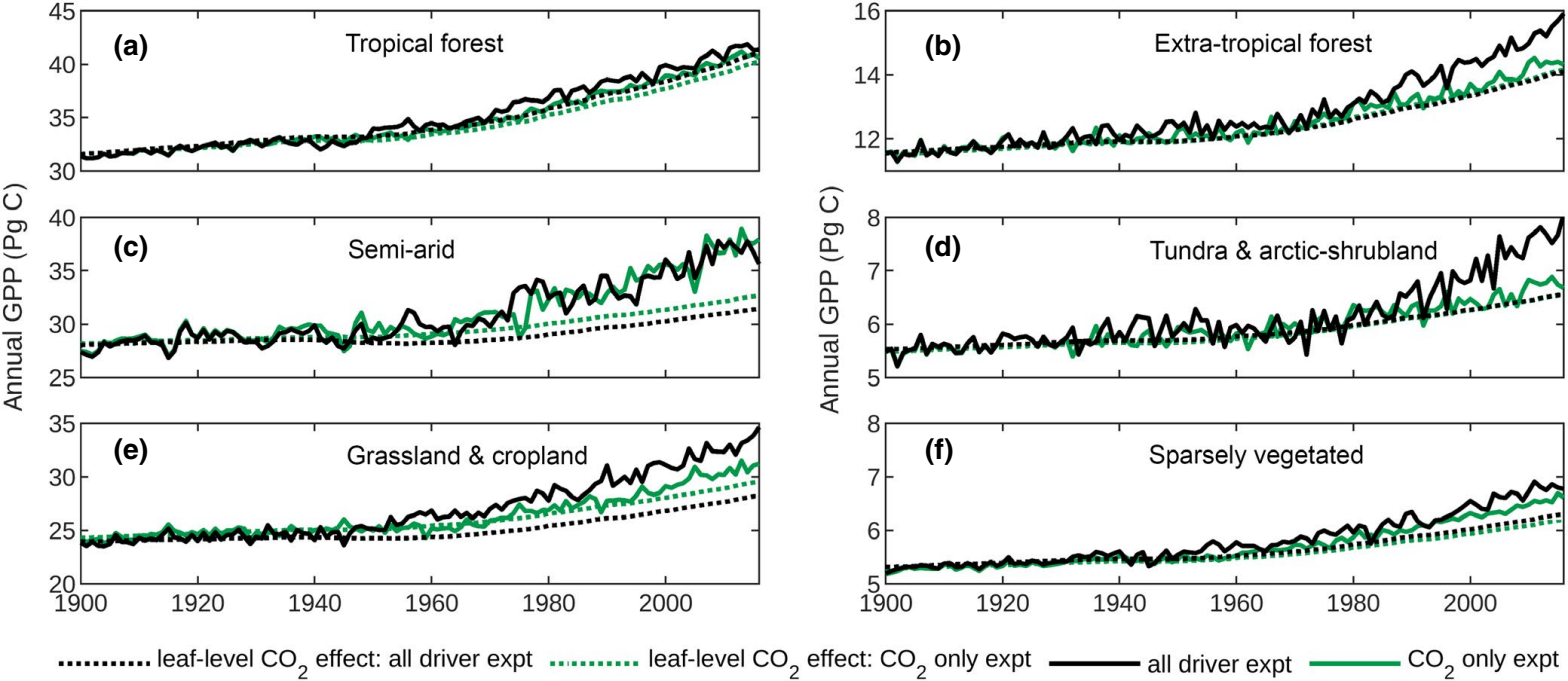
CO_2_ fertilization effect on gross primary production (GPP) by region. Regions correspond to land cover classification (Figure S3). Solid black line: full CABLE simulation with all drivers varying (“all driver expt”). Dotted black line: leaf‐level CO_2_ effect as a component of the all driver expt. Solid green line: CABLE simulation in which CO_2_ is varying, actual land cover assumed and recycled meteorology (1901–1930) is prescribed (“CO_2_ only expt”). Dotted green line: leaf‐level CO_2_ effect as a component of the CO_2_ only experiment. The difference between the green‐dotted and solid lines represents the CO_2_ greening effect. The difference between the solid green and black line represents the climate effect and *c_a_* × climate interaction effect on GPP

Two‐thirds of the global CO_2_‐greening effect is attributable to semiarid ecosystems. Over the (1982–2010) period, the 16% increase in semiarid GPP may be partitioned between leaf‐level (6%) and CO_2_‐greening (10%) components (C), in agreement with the 11% increase in foliage cover reported for the same period in warm semiarid regions where relative change in foliage cover may be equated with the CO_2_‐greening effect on GPP (Donohue, Roderick, Mcvicar, & Farquhar, 2013). In contrast, the tropical forest GPP (1982–2010) increases proportionately with *c_a_* (both 12%), almost entirely because of the leaf‐level effect (Figure 4a), in agreement with the increase (12%) inferred from tropical forest catchment water balance (Yang, Donohue, Mcvicar, Roderick, & Beck, 2016). Globally, the leaf‐level CO_2_ effect dominates total GPP increase (Figure 2a), meaning that vegetation greenness trends are not generally a direct proxy for GPP trends (see also De Kauwe, Keenan, Medlyn, Prentice, & Terrer, 2016).

**Figure 5:**
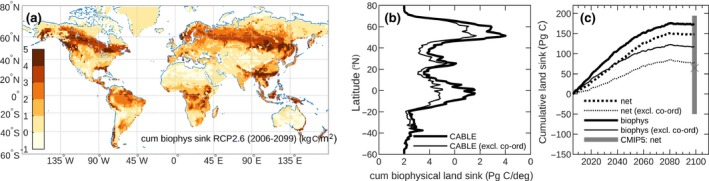
Projected cumulative land sink under Representative Concentration Pathway (RCP) 2.6 scenario. (a) Spatial distribution of cumulative biophysical (CO_2_ and climate‐driven) sink. (b) Latitudinal distribution of cumulative biophysical sink (full CABLE simulation, and with coordination of photosynthesis excluded). (c) Time series of projected (2006–2099) biophysical land sink (solid black lines) and net land sink (dotted black lines) relative to 2006 (full CABLE simulation (thick lines), and with coordination of photosynthesis excluded (thin lines). The range (bar) and average (cross) cumulative net land carbon sink projected by the CMIP5 carbon‐climate models (Ciais et al., 2013) are shown in grey

To explore the implications of a higher than expected CO_2_ fertilization effect for the future fate of the land carbon sink, we employed CABLE, constrained by observed global trends (Figure 1b–d) to project the net land sink (2006–2099) under a low emissions, moderate land use change scenario (RCP2.6). In this scenario, *c_a_* peaks at 440 ppm in 2050, global mean air temperature stabilizes at 2.4° above the 1900 level by 2040, carbon‐climate feedbacks are expected to be small (Ciais, Sabine, & Bala, 2013), and emissions from carbon‐stored permafrost are negligible (McGuire et al., 2018). Given these assumptions, we find that the terrestrial biosphere would continue to sequester carbon well into the second half of the century, leading to a cumulative net land sink (CO_2_, climate, and land use change driven) of 149 PgC by the end of the century, significantly larger than the mean of the CMIP5 carbon‐climate model ensemble under the RCP2.6 scenario (65 PgC; Ciais et al., 2013), but well within the among‐model spread of the same ensemble (−50 to +195 PgC; Figure 5c). Adjusting to include only biophysical effects (CO_2_ and climate‐driven sink), we estimate a cumulative biophysical sink of 174 PgC (spatial distribution; Figure 5a,b), equivalent to 17 years of anthropogenic CO_2_ emissions at current rates. This sink is large compared to the potential carbon uptake attainable by the recovery of existing secondary forests (117 Pg; Houghton & Nassikas, 2018), and 57 PgC larger than if a lower CO_2_ fertilization effect (simulated by CABLE disregarding coordination) is assumed (Figure 4c).

## 4 DISCUSSION

The magnitude of the simulated CO_2_ effect on electron transport‐limited photosynthesis (*A_j_*), the increase above this effect due to coordination, and the simulated reduction in *β* with *c_a_* emerging from our study are supported by other evidence. First, the historical CO_2_ fertilization effect on global GPP that is predicted by CABLE Excl. Coord. (*A* limited by *A_j_*) is comparable to that predicted by the diagnostic photosynthesis algorithm of Wang et al. (2017) that is unconstrained by coordination theory, and explicitly equates *A* with *A_j_* (Figure 2b). Furthermore, the same algorithm predicts a decline of *β* of (37%→19%) for a doubling of *c_a_* (400→800 ppm) at 25°C (Keenan et al., 2016). Using CABLE Excl. Coord., we find similar corresponding sensitivities (35%→17%), but much higher values of *β* (56%→34%) for full CABLE, in which coordination is allowed to occur (see also Figure S3a for latitudinal profiles of *β* at different *c_a_*). Second, by applying the full diagnostic photosynthesis algorithm of Wang et al. (2017) that is constrained by their alternate implementation of coordination theory, we confirm a large increase in *c_a_* sensitivity of historic global GPP when coordination is simulated, compared with the simulations in which *A* is limited to *A_j_* (Figure 2b). Third, as noted above, *β* decreases as *c_a_* increases, and this is due in part to a decline in maximum carboxylation capacity (*V*_cmax_) as relative investment of leaf nitrogen in electron transport increases such that coordination of photosynthesis is maintained. The simulated reduction in *V*_cmax_ agrees well with CO_2_‐acclimation effects on *V*_cmax_ inferred from free‐air CO_2_ enrichment (FACE) studies (~10% reduction for an increase in *c_a_* from 366 ppm to 567 ppm; Ainsworth & Rogers, 2007). We simulate a decrease of *V*_cmax_ */J*_max_ of around 20% (400–600 ppm *c_a_*), whereas a synthesis of FACE results reports a decrease of only 5% (Ainsworth & Rogers, 2007). The robustness of this average estimate is however questionable, since it relies on a fixed linear relationship between *J*_max_ and *V*_cmax_ (Ainsworth & Rogers, 2007). In fact, the proportionality between these quantities probably depends on nutrient status; as Wong (1979) first showed, cotton plants grown under low N (0.6 mmol/L nutrient solution) show a larger reduction of initial slope (*V*_cmax_) than do plants grown with plentiful N (24 mmol/L), but even in the latter case *V*_cmax_ */J*_max_ appears to decrease with elevated *c_a_*.

**Figure 6:**
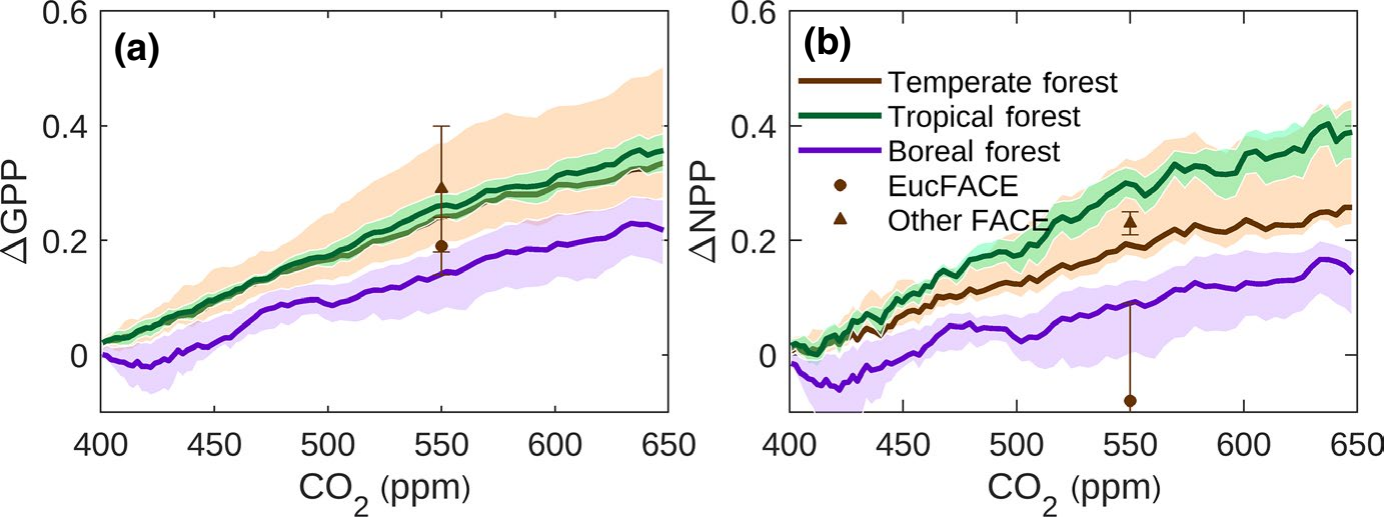
CO_2_ fertilization effect on gross primary production (GPP; a) and net primary production (NPP; b). The solid lines represent the median predictions of CABLE (fixed land use and climate, rising CO_2_ following the trajectory of RCP8.5) for each of temperate, tropical and boreal forests, referenced to a 400 ppm baseline. Shading represents 25th and 75th percentiles. Data points (triangles) are taken from meta‐analyses by Ainsworth and Rogers (2007; mean GPP response and standard error: results from two FACE experiments) and Norby et al. (2005; median NPP response and standard error: results from four FACE experiments). These experiments used average baseline and elevated concentrations of CO_2_ of 376 ppm and 550 ppm, respectively. Data points (circles) are taken from analysis of the EucFACE experiment by Ellsworth et al. (2017; baseline CO_2_: 400 ppm; elevated CO_2_ 550 ppm). In contrast to “Other FACE”, the EucFACE points refer to net (not gross) photosynthesis and aboveground (not total) NPP

Fourth, our simulated CO_2_ responses of GPP and net primary production (NPP; plant growth) in temperate forests globally agree well with the syntheses of data from FACE studies in temperate forests (Ainsworth & Rogers, 2007; Norby et al., 2005; Figure 6). EucFACE provides an exceptional example: here there is a negligible response of aboveground plant growth, probably because of strong phosphorus limitation (Ellsworth et al., 2017). Ecosystem models broadly reproduce the response of ecosystem NPP to elevated CO_2_ that is determined experimentally at FACE sites (Hickler et al., 2008; Zaehle et al. 2014). Differences in CO_2_ response between CABLE and other ecosystem models are mainly apparent in latitudes south of 15°N (tropics and savanna regions; see Figure S9, showing latitudinal contributions to each TRENDY model's global change in GPP since 1901). Hickler et al. (2008) likewise noted higher simulated responses of NPP to CO_2_ in the tropics than seen in temperate forest FACE sites.

Three other models in the TRENDYv6 ensemble simulate a centennial increase (1901–2010) of global GPP of ~30%, similar to that of CABLE and in agreement with the COS observation‐based estimate. These provide an opportunity to explore the alternative hypotheses to a higher‐than‐expected CO_2_ fertilization effect as explanations for the high increase in global GPP: (a) LPJ‐GUESS simulates very high land use change‐induced increase in Northern Hemisphere extratropical GPP (30–60°N), (b) JSBACH and JULES simulate high‐land use change and climate‐induced increases in GPP in the (−12 to −30°S) latitude band (Figure S9C‐D). Land use change is unlikely to induce the increases in GPP in this latitude band because agricultural land area over the last century has been dominated by clearing of tropical forest—a land use conversion that is expected to contribute negatively to trends in NPP (Nyawira, Nabel, Don, Brovkin, & Pongratz, 2016, table 4), and hence GPP. Climate‐induced increases in GPP simulated by JULES and JSBACH also appear to be unrealistic, based on apparent temperature and precipitation sensitivities derived from interannual variations in the global NLS (Figure S1b,c), which are known to be dominated by interannual variations in plant productivity in tropical forest and savanna regions (Ahlström et al., 2015). In particular, the apparent temperature sensitivity of JSBACH is positive whereas the atmospheric CO_2_ anomalies point to a negative relationship with the temperature (Figure S1b), while JULES exhibits an exaggerated sensitivity to the precipitation (Figure S1c). Thus, the high GPP increases in these models appear to stem partly from simulated climate sensitivities that are too high. Furthermore, the high‐relative changes are confounded with significant overestimates of GPP in these same latitude bands, compared with the observation‐based FLUXNET estimate (Figure S9a). This suggests that the contributions of land use change and climate alone in other models simulating a large GPP increase are likely to be too high. Our hypothesis of a large CO_2_ contribution, particularly in tropical forests and semiarid regions, is more compatible with the spatial distribution of GPP suggested by the FLUXNET data, and with the hydrological constraints (Yang et al., 2016) on GPP trends in tropical forests, as discussed above. Nonetheless, the role of agricultural expansion and intensification on global greening and GPP remains important and unresolved (Mueller et al., 2014; Zeng et al., 2014).

Our simulated latitudinal and regional distributions of trends in GPP and the NLS (Figure 3a,b; Figures S5–S8) are consistent with the observed increase in the ASC of CO_2_ in the Northern Hemisphere (56 ± 10% north of 45°N, 1960–2010; Graven et al., 2013), that is underestimated by current TBMs (Figure 1b), as previously reported (Graven et al., 2013; Thomas et al., 2016). Alternate explanations for the observed trend are increasing light‐use efficiency because of CO_2_ fertilization (Bastos et al., 2019; Piao et al., 2017; Thomas et al., 2016; Wenzel et al., 2016) or high‐latitude warming effects on biome distribution and plant productivity (Forkel et al., 2016). Warming effects emerged as important in simulations using the LPJmL TBM which accurately simulates the large observed ASC trend, (Forkel et al., 2016), but simulates a global GPP trend ( PgC/year^2^; 1970–2011; Forkel et al., 2016) that is less than half the corresponding CABLE‐simulated trend (0.53 ± 0.02 PgC/year^2^; 1970–2011). The CABLE trend is compatible with the COS‐based estimate (Campbell et al., 2017), whereas the LPJmL estimate appears too low. LPJmL is thus an example of a model that satisfies the regional ASC constraint, but not the global constraint on the trend in GPP. The high‐CO_2_ fertilization effect in CABLE, which is dominated by a leaf‐level effect in the Northern Hemisphere (Figure 4b,d), supports the hypothesis of increasing light‐use efficiency as a key driver of the amplitude of seasonal cycle increase. However, we note that this increase is highly dependent on lags between photosynthesis and ecosystem respiration and cannot be readily disentangled into warming versus *c_a_*‐driven trends in GPP.

The uncertain contribution of agricultural intensification to the increase in seasonal amplitude of CO_2_ in the Northern Hemisphere has been the subject of much debate (Gray et al., 2014; MacBean & Peylin, 2014; Zeng et al., 2014), but the following four points argue against agricultural intensification being a major contributor to global GPP increase: (a) the seasonal amplitude of CO_2_ in the Northern Hemisphere is sensitive to CO_2_ uptake and release north of 35°N (Graven et al., 2013) and not the whole globe, and land north of 35°N contributes less than 25% to global GPP (Figure S9). (b) Globally, it has been estimated that the combined effects of land use change and land management over the last century—including expansion of agricultural lands, and intensification by irrigation and fertilization—have had almost no impact on global NPP (Bondeau et al., 2007, figure 15f), although the effects on the seasonal cycle are nontrivial (Calle, Poulter, & Patra, 2019). (c) Expansion of agricultural land area over the last century has been dominated by clearing of tropical forest—a land use conversion that is expected to contribute negatively to trends in NPP (Nyawira et al., 2016). (d) Recent studies using multiple models and data sets have concluded that the effect of land use change and land management on the increase in seasonal amplitude of CO_2_ in the Northern Hemisphere is very small compared with the effects of CO_2_ fertilization and warming‐induced lengthening of growing season (Bastos et al., 2019; Piao et al., 2017). Bastos et al. (2019) identify Eurasia as the region contributing most to increasing seasonal amplitude of CO_2_—a region that is dominated by natural ecosystems and has experienced very little land use change (Verburg et al., 2015) over the last half‐century. A limitation of the study by Piao et al. (2017) is that it relies on an ensemble of models, many of which do not account for agricultural intensification. Bastos et al. (2019) address this limitation in their more recent study, by complementing the ecosystem model results with trend driver attribution using statistical models, confirming that the observed trends in seasonal amplitude of CO_2_ are not significantly related to agricultural intensification.

By reconciling multiple global‐scale observational constraints, we identified a CO_2_ fertilization effect on historical global GPP that is significantly higher than current estimates. Independent regional studies using amplitude of seasonal cycle data (Northern Hemisphere extra‐tropics; Wenzel et al., 2016) and catchment water balance (tropical forests; Yang et al., 2016) have also inferred larger CO_2_ fertilization effects than predicted by TBM ensembles. The causes of such model‐data discrepancies are poorly known, but biases associated with the representation of nutrient limitations on GPP have been invoked as one possible cause (Wenzel et al., 2016; Yang et al., 2016). Our results, that account for nitrogen‐cycle effects on ecosystem productivity, suggest that underprediction of GPP trends and CO_2_ responses is associated with a failure by current TBMs to account for plant coordination of photosynthesis. This finding is important for the future role of land carbon sinks, suggesting an underestimate by current models of potential CO_2_ removal under low‐emission scenarios consistent with the Paris Agreement targets.

## Data Availability

CABLE simulations used in this study can be found at: https://doi.org/10.5281/zenodo.3629955. The CABLE source code can be accessed after registration at https://trac.nci.org.au/trac/cable. Simulations in this work used Revision Number 4546.
