# Supplementary material for: Higher than expected CO2 fertilization inferred from leaf to global observations
Source: Glob Chang Biol. 2020 Feb 4;26(4):2390–402. doi: 10.1111/gcb.14950 (PMC7154678; doi:10.1111/gcb.14950)
Supplement: Supplementary file 1 [file GCB-26-2390-s001.docx]

Supplementary Information for

**Higher than expected CO_2_ fertilisation inferred from leaf to global observations**

Vanessa Haverd, Benjamin Smith, Josep G. Canadell, Matthias Cuntz, Sara Mikaloff-Fletcher, Graham Farquhar, William Woodgate, Peter R. Briggs, Cathy M. Trudinger

Corresponding author: Vanessa Haverd

Email: vanessa.haverd@csiro.au

**This PDF file includes:**

Figs. S1 to S9

Table S1

References for SI citations

**
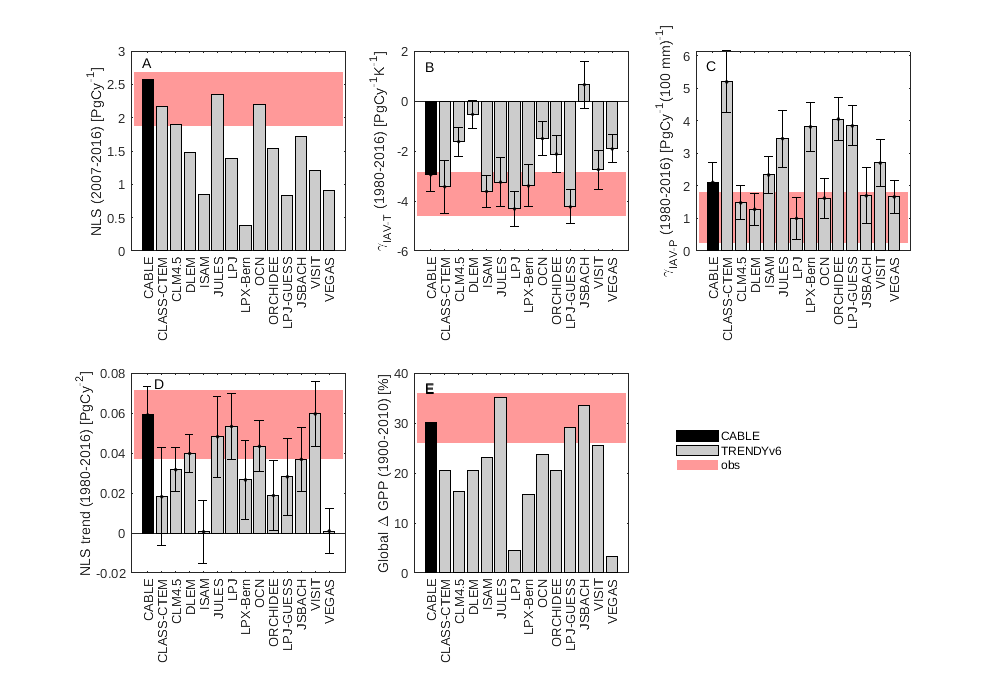
**

Fig. S1. Net land sink. Simulations by CABLE (black); TRENDYv6 (grey), observation based estimates (red shading). (A) mean NLS (2007 – 2016); (B) temperature sensitivity γ_IAV-T_ (1980-2016) derived from inter-annual variations according to Piao et al. (1). ); (C) precipitation sensitivity γ_IAV-P_ (1980-2016) derived from inter-annual variations according to Piao et al. (1). (D) NLS linear trend (1980-2016); (E) Increase in global GPP relative to 1900, simulated by CABLE (black) and TRENDYv6 (3, 10) (grey), and inferred from observations of [COS] (4) (red) .Observation shaded regions represent 1-σ estimates either side of the mean. The uncertainty on the mean NLS based on observations represents the combined 1-σ estimates on the GCP mass balance components. Error bars on γ_IAV_ represent standard errors on the temperature coefficient of the linear regression relating the global annual NLS (1980-2016) to mean annual temperature and precipitation in the −30° - +30° latitude band. Error bars on the NLS trend represent stand errors on the linear regression slope relating the global annual NLS to year (1980-2016).

**
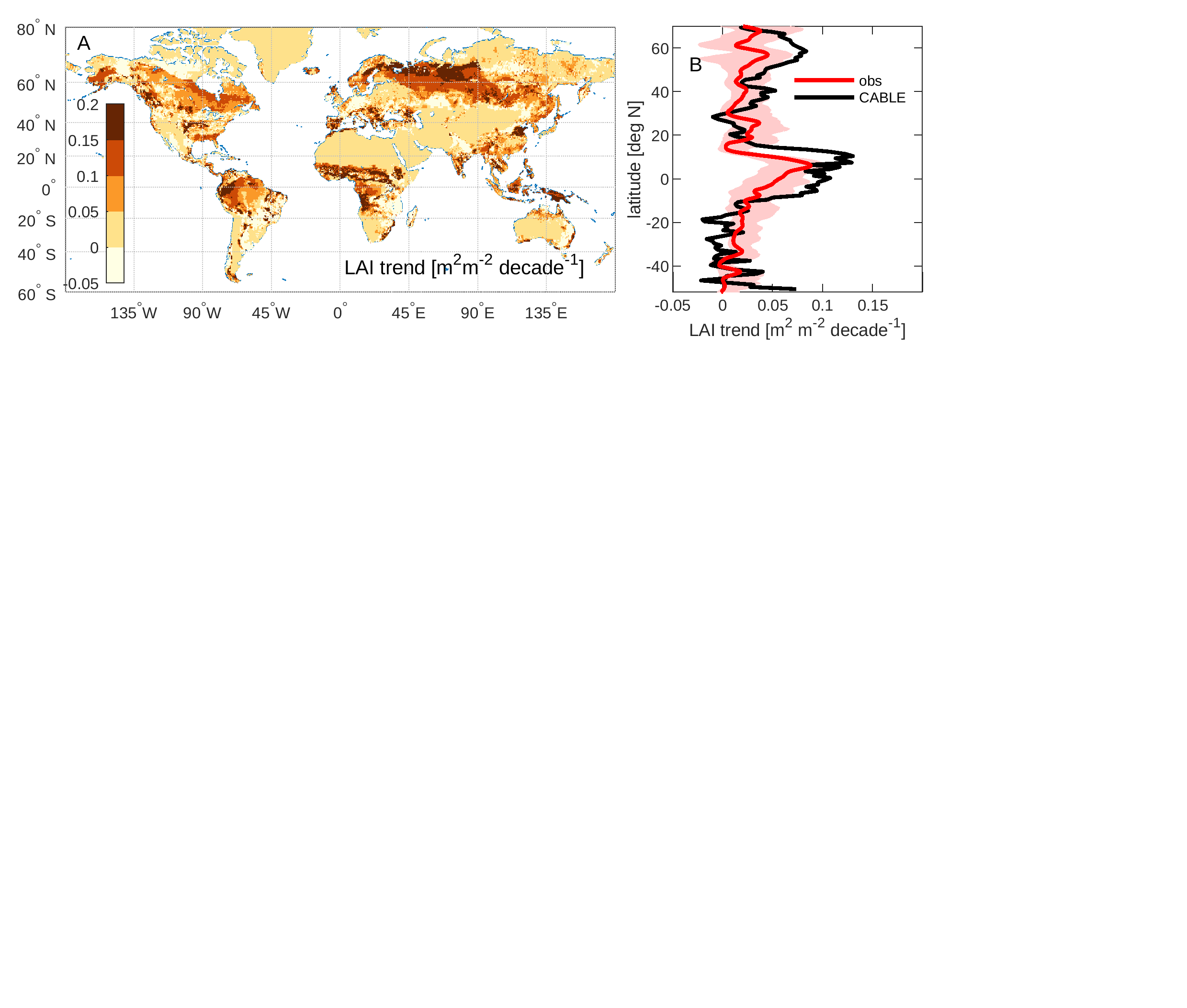
**

Fig. S2. Trends in leaf-area index (1981-2010). (A) Spatial distribution of simulated CABLE trends of mean annual LAI. (B) LAI trends averaged by latitude band as simulated by CABLE (black) and median trends derived from GIMMS-3g satellite-based observations with inter-quartile range shaded (red). GIMMS-3g data reproduced with permission from Fig. 3b of Forzieri et al. (2). Data south of 52 °S omitted due to insignificant land area.


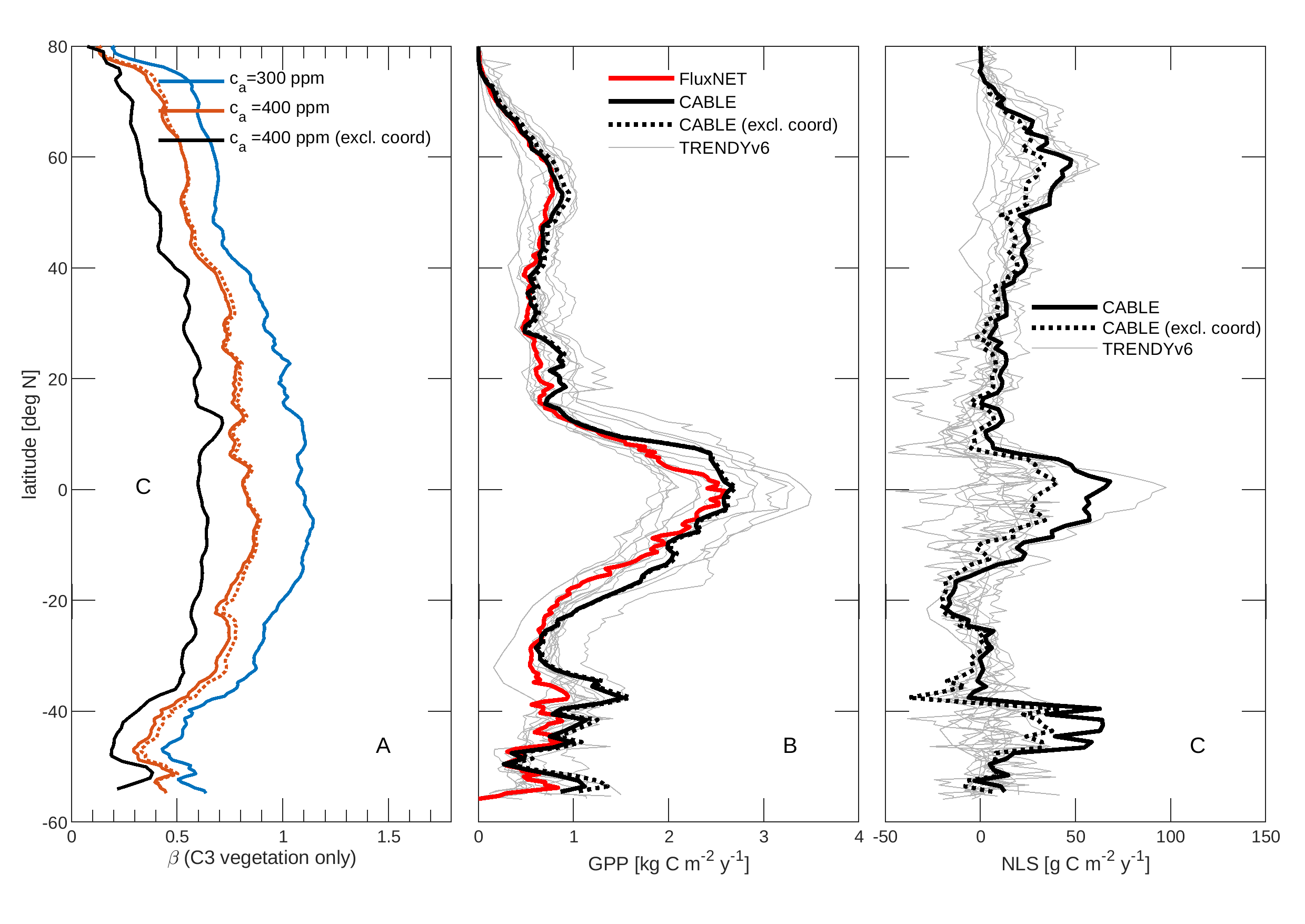


Fig. S3. Latitudinal profiles of β (C_3_ vegetation only), GPP and NLS. (A) A_n_-weighted *β* for: (blue) *c_a_* = 300 ppm (1911), (solid red) and *c_a_* = 400 ppm (2015 *c_a_*; 1911 climate), (dotted red) *c_a_* = 400 ppm (2015 *c_a_*; 2015 climate), and (black) *c_a_* = 400 ppm (2015 *c_a_*; 1911 climate; coordination excluded, i.e. photosynthesis limited exclusively by electron transport). The increase in *c_a_* from 300 ppm to 400 ppm leads to a latitudinally-averaged reduction in β of 0.20 ± 0.06, while higher temperatures in 2015 (relative to 1911) increase β by 0.02 ± 0.01. The shift from coordination to exclusive electron transport limitation leads to a latitudinally-averaged reduction in β of 0.18 ± 0.05. (B) Area-based GPP (1982-2011): upscaled eddy flux data (3) (red); CABLE (solid black); CABLE with coordination excluded (dotted black); TRENDYv6 (grey). (C) Area-based NLS: CABLE (solid black); CABLE with coordination excluded (dotted black); TRENDYv6 (grey).

Fig. S4. Land-Cover classification for use in regional attribution of trends in GPP and the land carbon sink. Reproduced with permission from Ahlström et al. (4).


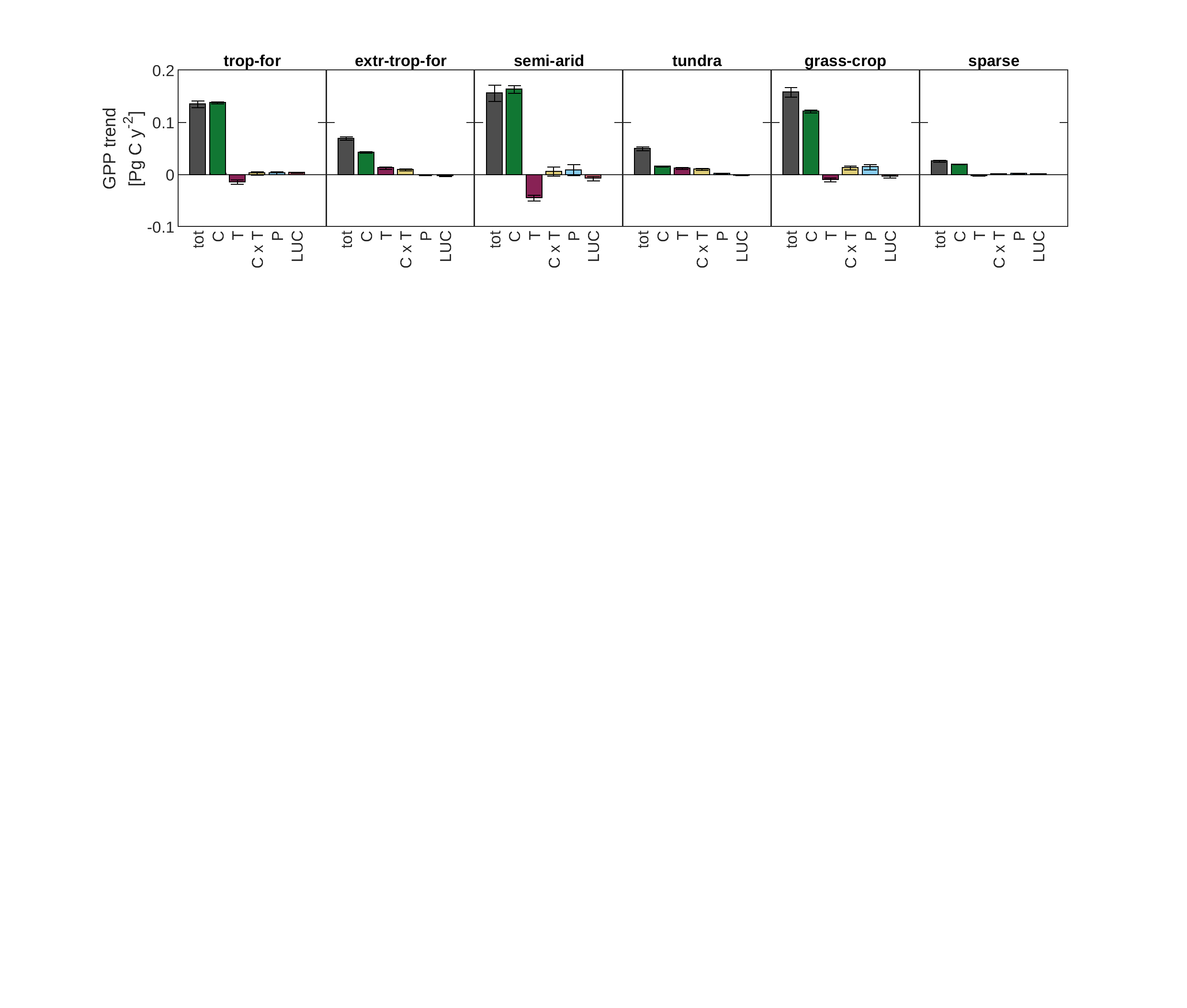


Fig. S5. Attribution of GPP trend (1980-2016) to regions and drivers. Land-cover classes correspond to Fig S3. Driver attribution is achieved by factorial experiments in which only one or a pair of drivers are varied while all others are fixed (Methods). Tot = Total effect; C = *c_a_* only; T = temperature; C × T = *c_a_* × Temperature interaction; P = precipitation; LUC = land-use change. Only major drivers are isolated. Additional drivers (nitrogen deposition; specific humidity; wind speed; incoming short and long-wave radiation) and their interactions are not isolated but may contribute to the total effect.


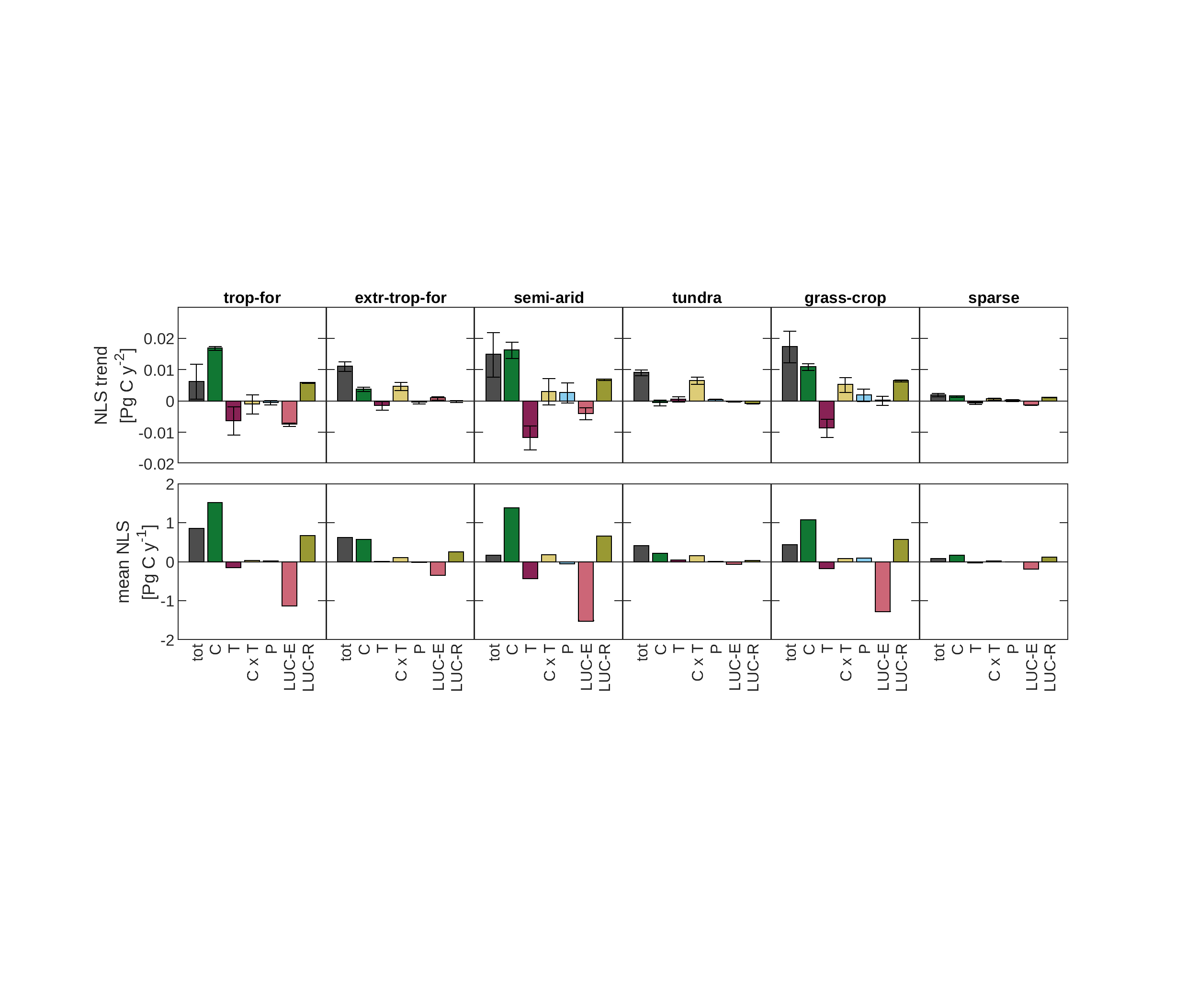


Fig. S6. Attribution of NLS trend (1980-2016) and mean NLS (2007-2016) to regions and drivers. Driver attribution is achieved by factorial experiments in which only one or a pair of drivers are varied while all others are fixed (Methods). Tot = Total effect; C = *c_a_* only; T = temperature; C × T = *c_a_*-Temperature interaction; P = precipitation; LUC-E = land-use change gross emissions; LUC-R = land-use change regrowth. Only major drivers are isolated. Additional drivers (nitrogen deposition; specific humidity; wind speed; incoming short and long-wave radiation) and their interactions are not isolated but contribute to the total effect.


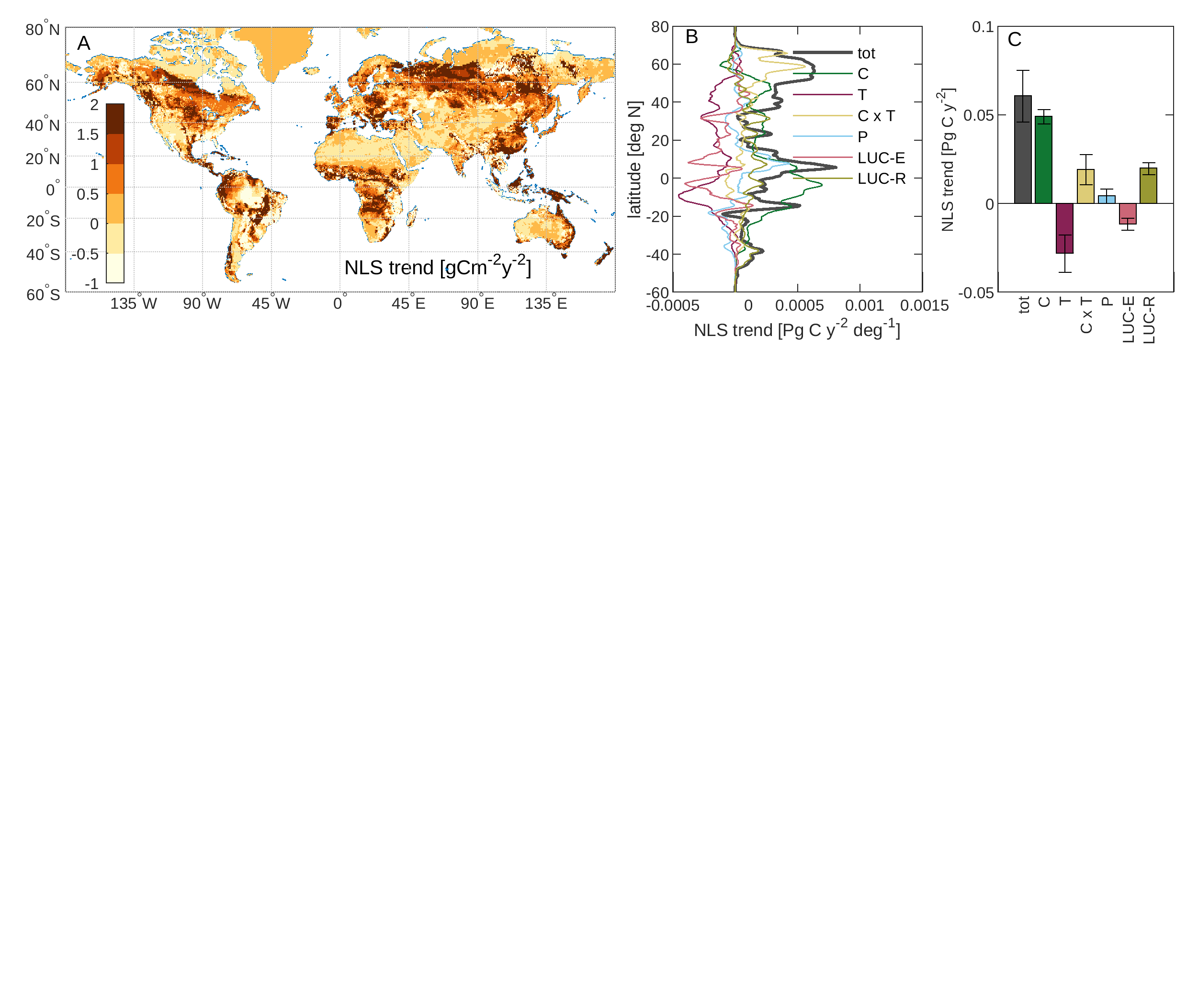


Fig. S7. Attribution of NLS trend (1980-2016).

**
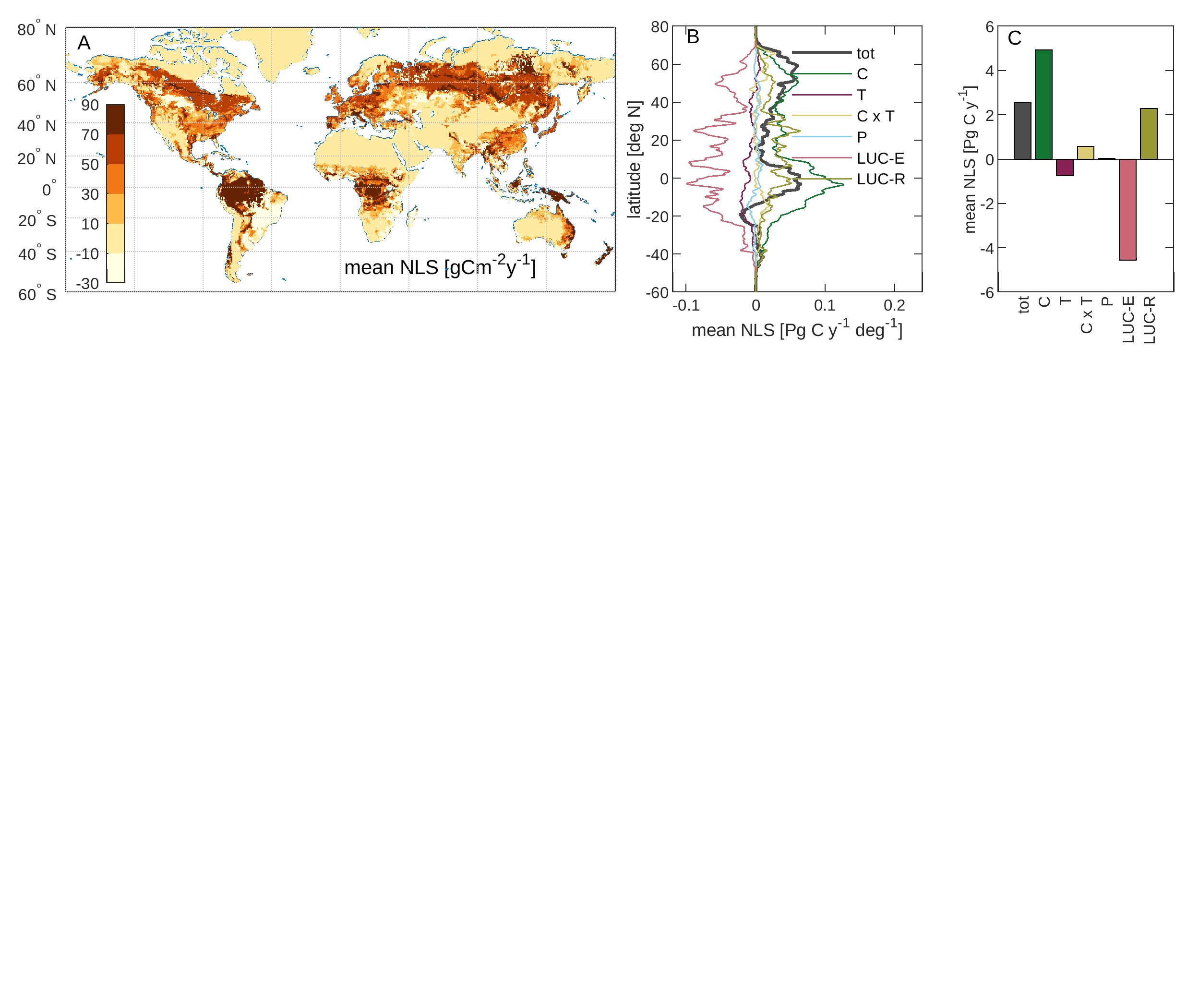
**

Fig. S8. Attribution of mean NLS (2007-2016).


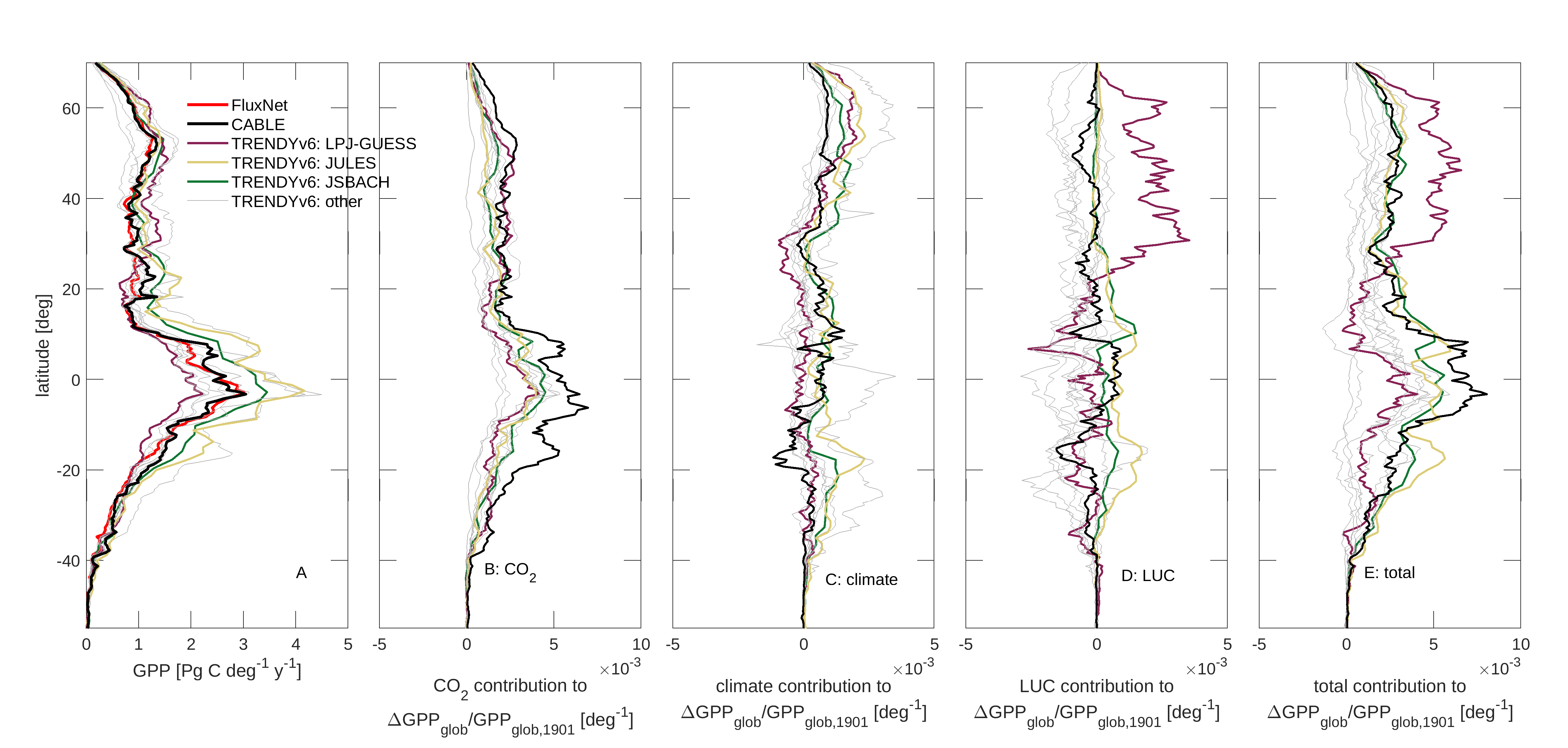


Fig. S9. Driver-attribution of simulated latitudinal contributions to relative change in global GPP (1901-2010). Simulations by CABLE and TRENDYv6. LPJ-GUESS, JULES and JSBACH simulations are distinguished from the rest of the ensemble because they have total relative global GPP increases of ~30%, consistent with the COS observation-based estimate. (A) latitudinal distribution of GPP. (B) CO_2_ contribution to relative change in global GPP, derived from CO_2_-only model experiment. (C) Climate contribution, derived from CO_2_/climate simulation minus CO_2_ only experiment (D) Land-use change contribution, derived from all-driver simulation minus CO_2_/climate simulation.

Table S1. TRENDYv6 model names and references.

| **Model** | **Reference** |
| --- | --- |
| CLASS-CTEM | Melton and Arora (2016) (6) |
| CLM4.5(BGC) | Oleson et al. (2013) (7) |
| DLEM | Tian et al. (2015) (8) |
| ISAM | Jain et al. (2013) (9) |
| JSBACH | Reick et al. (2013) (10) |
| JULES | Clark et al. (2011) (11) |
| LPJ | Sitch et al. (2003) (12) |
| LPX | Keller et al. (2017) (13) |
| LPJ-GUESS | Smith et al. (2014) (14) |
| OCN | Zaehle and Friend (2010) (15) |
| ORCHIDEE | Krinner et al. (2005) (16) |
| VEGAS | Zeng et al. (2005) (17) |
| VISIT | Kato et al. (2013) (18) |

**References**

1. Piao S et al. (2013) Evaluation of terrestrial carbon cycle models for their response to climate variability and to CO_2_ trends. *Global Change Biol* 19:2117–2132.
2. Forzieri G, Alkama R, Miralles DG, Cescatti A (2017) Satellites reveal contrasting responses of regional climate to the widespread greening of Earth. *Science* 356:1180–1184.
3. Jung M et al. (2011) Global patterns of land-atmosphere fluxes of carbon dioxide, latent heat, and sensible heat derived from eddy covariance, satellite, and meteorological observations. *J Geophys Res-Biogeo* 116:G00J07.
4. Ahlström A et al. (2015) The dominant role of semi-arid ecosystems in the trend and variability of the land CO_2_ sink. *Science* 348:895–899.
5. Wang H et al. (2017) Towards a universal model for carbon dioxide uptake by plants. *Nature Plants* 3:734–741.
6. Melton J R, Arora VK (2016) Competition between plant functional types in the Canadian Terrestrial Ecosystem Model (CTEM) v. 2.0. *Geosci Model Dev* 9:323–361.
7. Oleson K et al. (2013) Technical Description of version 4.5 of the Community Land Model (CLM), *National Center of Atmospheric Research (NCAR)*, Boulder, Colorado, USA.
8. Tian H et al. (2015) North American terrestrial CO_2_ uptake largely offset by CH_4_ and N_2_O emissions: toward a full accounting of the greenhouse gas budget. *Clim Change* 129:413–426.
9. Jain A K, Meiyappan P, Song Y, House JI (2013) CO_2_ emissions from land‐use change affected more by nitrogen cycle, than by the choice of land‐cover data. *Global Change Biol* 19:2893–2906.
10. Reick CH, Raddatz T, Brovkin V, Gayler V (2013) Representation of natural and anthropogenic land cover change in MPI‐ESM. *J Adv Model Earth Sys* 5:459–482.
11. Clark D B et al. (2011) The Joint UK Land Environment Simulator (JULES), model description – Part 2: Carbon fluxes and vegetation dynamics. *Geosci Model Dev* 4:701–722.
12. Sitch S et al. (2003) Evaluation of ecosystem dynamics, plant geography and terrestrial carbon cycling in the LPJ dynamic global vegetation model. *Global Change Biol* 9:161–185.
13. Keller KM et al. (2017) 20^th^ century changes in carbon isotopes and water-use efficiency: tree-ring-based evaluation of the CLM4.5 and LPX-Bern models. *Biogeosciences* 14:2641–2673.
14. Smith B et al. (2014) Implications of incorporating N cycling and N limitations on primary production in an individual-based dynamic vegetation model. *Biogeosciences* 11:2027–2054.
15. Zaehle S and Friend DA (2010) Carbon and nitrogen cycle dynamics in the O‐CN land surface model: 1. Model description, site‐scale evaluation, and sensitivity to parameter estimates. *Global Biogeochem Cyc* 24:GB1005.
16. Krinner G et al. (2005) A dynamic global vegetation model for studies of the coupled atmosphere‐biosphere system. *Global Biogeochem Cyc* 19:GB1015.
17. Zeng N, Qian H, Roedenbeck C, Heimann M (2005) Impact of 1998–2002 midlatitude drought and warming on terrestrial ecosystem and the global carbon cycle. *Geophys Res Lett* 32: L22709.
18. Kato E, Kinoshita T, Ito A, Kawamiya M, Yamagata Y (2013) Evaluation of spatially explicit emission scenario of land-use change and biomass burning using a process-based biogeochemical model. *J Land Use Science* 8:104–122.
19. Ainsworth EA & Rogers A (2007) The response of photosynthesis and stomatal conductance to rising [CO_2_]: mechanisms and environmental interactions. *Plant, Cell Environ.* 30(3):258-270.
20. Norby RJ*, et al.* (2005) Forest response to elevated CO2 is conserved across a broad range of productivity. *Proceedings of the National Academy of Sciences of the United States of America* 102(50):18052-18056.
21. Ellsworth David S*, et al.* (2017) Elevated CO_2_ does not increase eucalypt forest productivity on a low-phosphorus soil. *Nature Climate Change* 7:279.
22. Heimann M & Körner S (2005) The global atmospheric tracer model TM3. . in *Technical Reports* (Max-Planck-Institut für Biogeochemie), p 131.
23. Kalnay E*, et al.* (1996) The NCEP/NCAR 40-Year Reanalysis Project. *Bulletin of the American Meteorological Society* 77(3):437-472.
24. Graven HD*, et al.* (2013) Enhanced Seasonal Exchange of CO_2_ by Northern Ecosystems Since 1960. *Science* 341(6150):1085.
25. Keeling CD, Harris TB, & Wilkins EM (1968) Concentration of atmospheric carbon dioxide at 500 and 700 millibars. *Journal of Geophysical Research* 73(14):4511-4528.
26. Wofsy SC (2011) HIAPER Pole-to-Pole Observations (HIPPO): fine-grained, global-scale measurements of climatically important atmospheric gases and aerosols. *Philosophical Transactions of the Royal Society A: Mathematical, Physical and Engineering Sciences* 369(1943):2073.
27. Thomas RT*, et al.* (2016) Increased light-use efficiency in northern terrestrial ecosystems indicated by CO_2_ and greening observations. *Geophys. Res. Lett.* 43(21):11,339-311,349.
28. Wenzel S, Cox PM, Eyring V, & Friedlingstein P (2016) Projected land photosynthesis constrained by changes in the seasonal cycle of atmospheric CO_2_. *Nature* 538(7626):499-501.
29. Koven CD*, et al.* (2013) The effect of vertically resolved soil biogeochemistry and alternate soil C and N models on C dynamics of CLM4. *Biogeosciences* 10(11):7109-7131.
30. Keeling CD*, et al.* (2001) Exchanges of atmospheric CO_2_ and ^13^CO_2_ with the terrestrial biosphere and oceans from 1978 to 2000. I. Global aspects. in *SIO Reference Series* (Scripps Institution of Oceanography, San Diego), p 88.
31. Haverd V*, et al.* (2018) A new version of the CABLE land surface model (Subversion revision r4601) incorporating land use and land cover change, woody vegetation demography, and a novel optimisation-based approach to plant coordination of photosynthesis. *Geosci. Model Dev.* 11(7):2995-3026.
32. Walker AP*, et al.* (2014) The relationship of leaf photosynthetic traits – Vcmax and Jmax – to leaf nitrogen, leaf phosphorus, and specific leaf area: a meta-analysis and modeling study. *Ecology and Evolution* 4(16):3218-3235.
